# Solution-Deposited Solid-State Electrochromic Windows

**DOI:** 10.1016/j.isci.2018.11.014

**Published:** 2018-11-10

**Authors:** Wei Cheng, Marta Moreno-Gonzalez, Ke Hu, Caroline Krzyszkowski, David J. Dvorak, David M. Weekes, Brian Tam, Curtis P. Berlinguette

**Affiliations:** 1 Department of Chemistry, The University of British Columbia, 2036 Main Mall, Vancouver, BC V6T 1Z1, Canada; 2 Department of Chemical and Biological Engineering, The University of British Columbia, 2360 East Mall, Vancouver, BC V6T 1Z3, Canada; 3 Stewart Blusson Quantum Matter Institute, The University of British Columbia, 2355 East Mall, Vancouver, BC V6T 1Z4, Canada

**Keywords:** materials science, coatings, energy materials

## Abstract

Commercially available electrochromic (EC) windows are based on solid-state devices in which WO_3_ and NiO_x_ films commonly serve as the EC and counter electrode layers, respectively. These metal oxide layers are typically physically deposited under vacuum, a time- and capital-intensive process when using rigid substrates. Herein we report a facile solution deposition method for producing amorphous WO_3_ and NiO_x_ layers that prove to be effective materials for a solid-state EC device. The full device containing these solution-processed layers demonstrates performance metrics that meet or exceed the benchmark set by devices containing physically deposited layers of the same compositions. The superior EC performance measured for our devices is attributed to the amorphous nature of the NiO_x_ produced by the solution-based photodeposition method, which yields a more effective ion storage counter electrode relative to the crystalline NiO_x_ layers that are more widely used. This versatile method yields a distinctive approach for constructing EC windows.

## Highlights

• Amorphous WO_3_ and NiO_x_ films are produced by a solution-deposition method
• The WO_3_ and NiO_x_ films are assembled into a solid-state electrochromic device
• The solid-state device exhibits state-of-the-art electrochromic performance
• Amorphous NiO_x_ is a superior counter electrode material compared with crystalline NiO_x_

## Graphical Abstract

**Figure:**
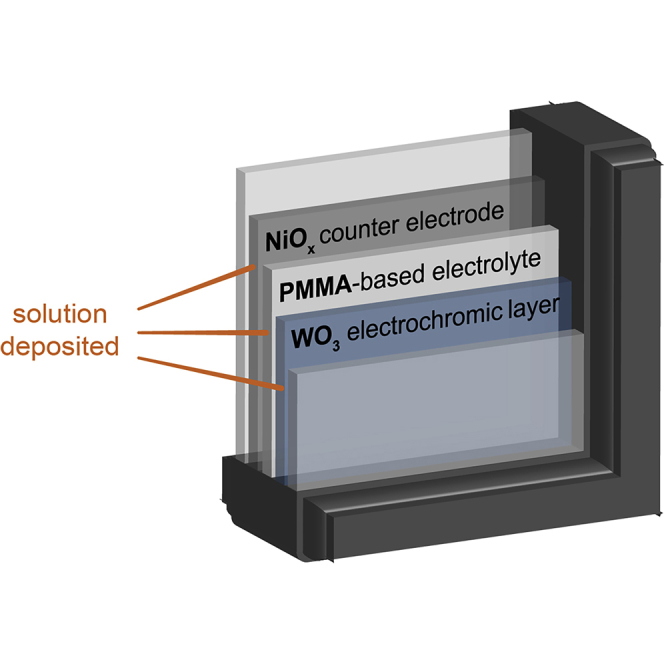

## Introduction

Electrochromic (EC) windows (also known as smart or dynamic windows) undergo changes in light transmittance in response to an applied voltage, enabling the dynamic control of daylight and solar heat passing through buildings (Granqvist, 2006, Granqvist, 2012a, Runnerstrom et al., 2014). This technology can provide both indoor thermal and visual comfort for building occupants while improving building energy efficiency by as much as 20% (Granqvist, 2012a). These features have prompted substantial investment into deploying EC windows at scale, but the high price of such windows ($1,000/m^2^ *c.f*. ∼$150/m^2^ for a regular window (Smart windows cost, 2016)) has prevented their widespread use. A significant fraction of the cost is imbedded in the vacuum environment required to sputter EC window layers. This process can require a substantial capital outlay, and the long residence times needed to fabricate the key layers on rigid substrates also preclude rapid throughput (Garg et al., 2005). These factors present the impetus to develop solution-based deposition methodologies to reduce the costs associated with fabricating EC windows (Barile et al., 2017, Cai et al., 2016a, Llordés et al., 2016).

**Figure 1:**
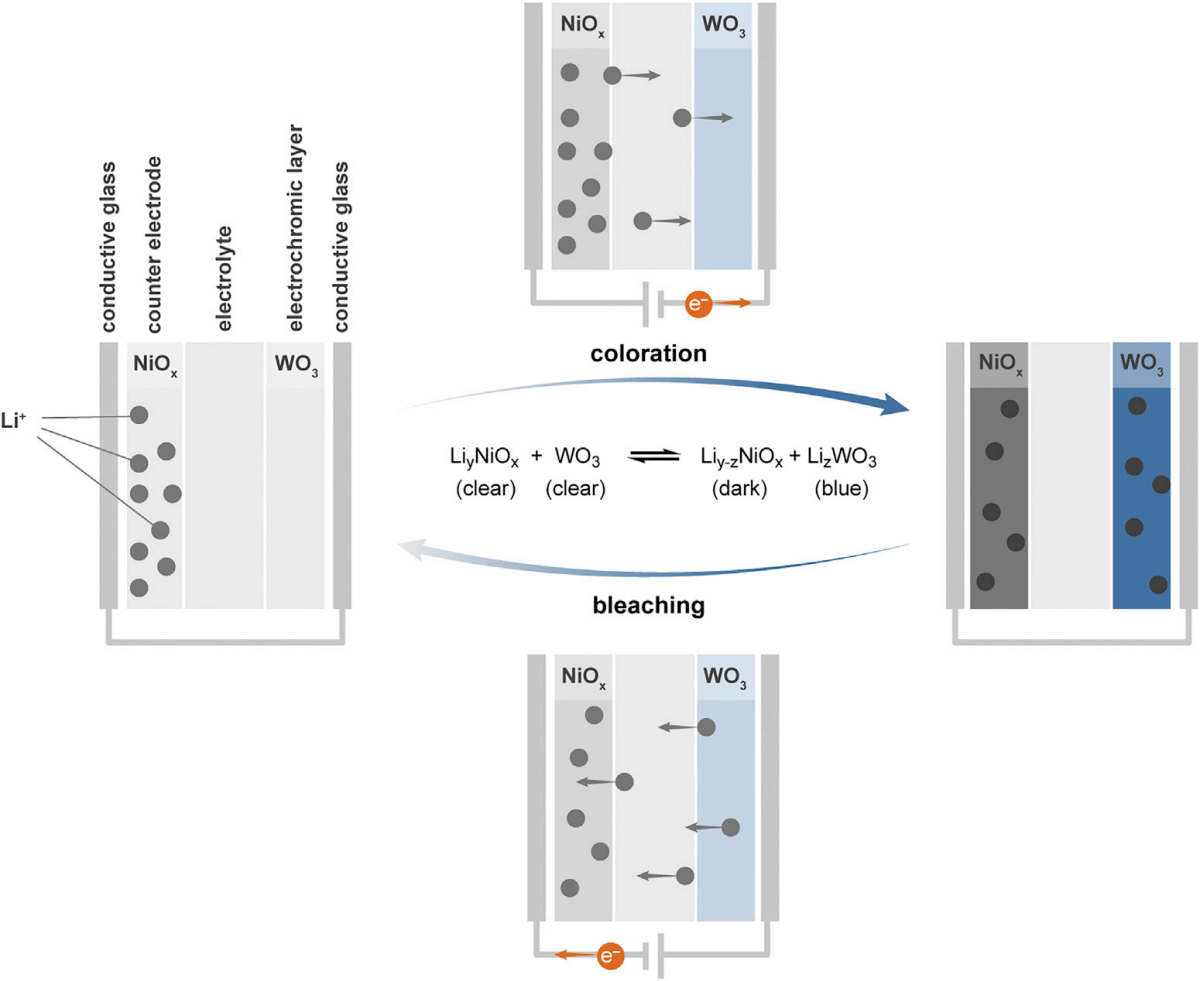
Working Principles for an Electrochromic Device Scheme outlining the coloration and bleaching process of an EC device with a WO_3_ electrochromic layer and NiO_x_ counter electrode layer sandwiched between two transparent conductive glass substrates. The pristine device is formed by transparent Li_y_NO_x_ and WO_3_ layers and an interstitial electrolyte. Applying a forward bias to the electrochromic layer of the device injects electrons into WO_3_, which are then compensated by Li^+^ migrating from the counter electrode through the electrolyte to yield colored Li_x_WO_3_. The simultaneous extrusion of Li^+^ from Li_y_NiO_x_ enables the formation of colored Li_y-z_NO_x_. The bleaching process is obtained by applying a reverse bias, yielding the initial Li_y_NiO_x_ and WO_3_ transparent layers. The coloration and bleaching processes are summarized by the overall reversible reaction shown in the Scheme.

A conventional EC window consists of an EC layer, an electrolyte layer, and an ion storage counter electrode sandwiched between two transparent conducting layers (Figure 1). Thin films of WO_3_ and NiO_x_ are widely used as the respective EC and counter electrode layers in commercial EC windows (Gillaspie et al., 2010, Niklasson and Granqvist, 2006). Both of these layers contribute to reversible color switching in response to an electrical bias (Figure 1). The NiO_x_ layers are lithiated to form Li_y_NiO_x_ before device assembly to provide a source of intercalating Li^+^ during reduction (coloration) of WO_3_. A voltage applied to the assembled device drives coloration of both metal oxide layers as Li^+^ migrates into the WO_3_ layer. The ion-conducting and electrically insulating electrolyte layer serves to shuttle Li^+^ between the metal oxide layers and prevent short circuiting of the device. Liquid electrolytes are common in academic studies, but solid polymer-based electrolytes are used in commercial systems to satisfy safety and sealing issues (Granqvist, 2012b, Thakur et al., 2012).

The EC behavior of these layers is exquisitely sensitive to impurities, film defects, thickness, porosity, and crystallinity (Cai et al., 2016b, Granqvist, 2014, Lee et al., 2006, Scherer and Steiner, 2013). These factors therefore impose very stringent conditions on how EC windows can be manufactured. Physical vacuum deposition has emerged as the most reliable and scalable method of producing uniform and optical-quality metal oxide films of variable thicknesses (Thummavichai et al., 2017). Notwithstanding, the metal oxide semiconductor industry teaches that manufacturing methods progressively transition from vacuum to solution-processing methods to reduce costs (Yu et al., 2016). The ability to solution process at ambient temperatures and pressures offers the opportunity to reduce energy consumption and capital equipment while also reducing processing times (Llordés et al., 2016). Moreover, the avoidance of high vacuum lessens the safety concerns associated with handling potentially hazardous materials (e.g., lithium-containing chemicals). There are several reports of solution-based methods for processing EC or counter electrode layers, including electrodeposition (Baeck et al., 2003, Cai et al., 2016b), chemical bath deposition (Ristova et al., 2002, Vidales-Hurtado and Mendoza-Galván, 2008, Xia et al., 2008), sol-gel methods (Livage and Ganguli, 2001), and spray pyrolysis (Kamal et al., 2005, Tenent et al., 2010). However, none of these methods are well suited for synthesizing *both* WO_3_ and NiO_x_ films. Consequently, these layers are evaluated in electrochemical half-cells or in devices in which the working or counter electrode is physically deposited (Jiao et al., 2003, Srivastava et al., 2005, Zhang et al., 2009). These observations motivated us to build an EC device wherein all layers are solution-processed.

We demonstrate herein the use of a “photodeposition” method (Cheng et al., 2018, Smith et al., 2013) to solution-process both the WO_3_ and NiO_x_ EC layers of an EC device with a solution-deposited poly(methyl methacrylate) (PMMA)-based electrolyte. To our knowledge, this is the first report of a solid-state EC device containing internal layers that are fully solution-processable at low temperature. The performance of our laboratory-scale solid-state EC devices meets or exceeds current literature benchmarks in terms of optical modulation (ΔT_633 nm_; measured as the difference in light transmittance between the fully colored and bleached states at λ = 633 nm), switching time for coloration (t_color_; defined by the time required to reach 90% of a transmittance change from the fully bleached to fully colored state) and bleaching (t_bleach_), and coloration efficiency (CE; the change in optical density acquired by injection of charge per unit area; Table 1). We also demonstrate the superior performance of amorphous thin film materials in EC windows by directly comparing amorphous and crystalline NiO_x_ counter electrodes in the same device architecture and produced by analogous solution-based methods. This study presents an opportunity to harness low-cost solution-processing methods for the production of high quality EC devices.

**Table 1:** Performance Parameters of Our Solid-State EC Devices in Comparison with That of Solid-State Devices Reported in the Literature

Counter Electrode Material	Phase	Deposition Method	ΔT (%)	t_color_ (s)	t_bleach_ (s)	CE (cm^2^/C)	Reference
NiO porous film	Crystalline	Chemical bath deposition	55	10	20	87.2	Zhang et al., 2009^a^
NiO nanoparticle film	Crystalline	Inkjet printing	75	10	13	131.9	Cai et al., 2016a^b^
NiO_x_ film	Crystalline	Magnetron sputtering	52	5	2	–	Liu et al., 2016^c^
**NiO**_x_**film**	**Amorphous**	**Photodeposition**	**60**	**4**	**6**	**141**	**this work**^d^
**NiO**_x_**film**	**Crystalline**	**Photodeposition**	**26**	**78**	**17**	**72**	**this work**^d^

All devices use WO_3_ as the electrochromic layer, NiO_x_ as the counter electrode, and a polymer-based electrolyte.

a Measured at 633 nm over the −2.5 to +2.5 V potential range.

b Measured at 550 nm over the −2.5 to +2.5 V potential range.

c Measured at 550 nm over the −1.8 to +1.8 V potential range.

d Measured at 633 nm over the −2.1 to +2.1 V potential range.

## Results and Discussion

### Electrode Synthesis and Device Assembly

**Figure 2:**
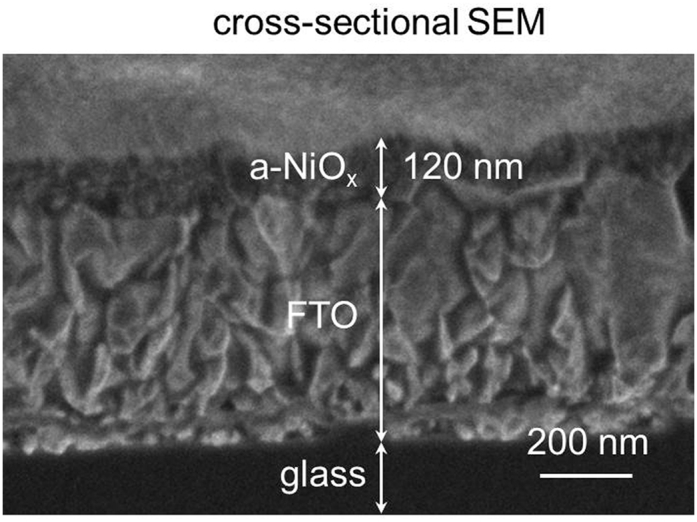
SEM Characterization of Amorphous NiO_x_ Films Cross-sectional SEM images of five-layer a-NiO_x_ films produced by photodeposition followed by annealing at 200°C for 1 hr. Cross-sectional SEM images were acquired on fresh edges of cleaved samples at a tilt angle of 52°. See also Figures S1–S4, S6, and S7.

Amorphous tungsten oxide (a-WO_3_) films were prepared using a photodeposition methodology known to yield amorphous metal oxide layers (Cheng et al., 2018, He et al., 2017, Smith et al., 2013). Briefly, a solution of WCl_6_ in 2-propanol was spin-cast onto a fluorine-doped tin oxide (FTO) substrate and the resultant precursor film was irradiated with UV light (λ_max_ = 185 nm) to form a metal oxide film. Characterization of the films by top-view and cross-sectional scanning electron microscopy (SEM) was consistent with the successful formation of porous a-WO_3_ films of ∼600 nm thickness (Figure S1). NiO_x_ films were prepared following a similar procedure: aqueous solutions of 0.25 M NiCl_2_ were spin-cast onto an FTO substrate followed by UV irradiation (λ_max_ = 185 nm) for 8 hr. The complete liberation of chlorine from the precursor film and the formation of NiO_x_ was confirmed by X-ray fluorescence and X-ray photoelectron spectroscopy analyses (Figure S2). The X-ray diffraction (XRD) pattern of the as-prepared NiO_x_ films showed a broad reflection centered at 2θ = 18° indexed to the (001) facets of α-Ni(OH)_2_ (Smith et al., 2016), together with the reflections corresponding to the FTO substrate (Figure S3). This broad reflection was no longer observed, and no additional reflections appeared after annealing the NiO_x_ films at 200°C for 1 hr, which denotes the successful formation of amorphous films (Figure S3). The thickness of a-NiO_x_ films prepared by spin casting and UV treating five layers of NiCl_2_ precursor on the FTO substrate before the annealing step was determined to be 120 nm by cross-sectional SEM (Figure 2). Cross-sectional and top-view SEM images (Figures 2 and S4A) show that the NiO_x_ films follow the contour of FTO and have smooth uniform surfaces. Crystalline NiO_x_ (c-NiO_x_) films were also obtained by annealing the as-deposited NiO_x_ films at 400°C for 1 hr. The resulting XRD pattern shows well-defined reflections corresponding to crystalline cubic NiO (Figure S3). Five-layer c-NiO_x_ films show smooth uniform surfaces with a thickness of 105 nm (Figures S4B and S4C).

Solid-state EC devices with active areas of ∼2 cm^2^ were assembled using a-WO_3_ films coated on FTO glass as the working electrode, either a- or c-NiO_x_ films deposited on FTO glass as the counter electrode, and a PMMA-based gel as the electrolyte. NiO_x_ counter electrodes were lithiated before device assembly by submerging the films in 1 M LiClO_4_ propylene carbonate and applying a potential of −1.5 V (vs Ag/AgCl) across the cell for 5 min. The PMMA-based gel electrolyte was drop-cast directly onto the a-NiO_x_ electrode and contained within a silicone spacer with a thickness of 1 mm. The device was completed with the a-WO_3_ working electrode and sealed with epoxy glue.

### Electrochromic Performance of Solid-State Devices

**Figure 3:**
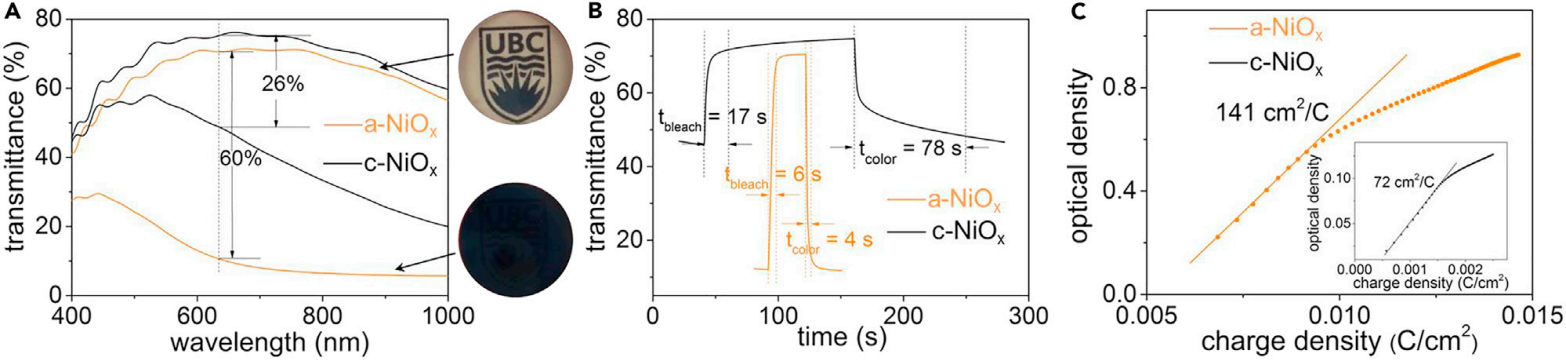
Amorphous NiO_x_ Layers Make Superior Counter Electrodes (A) Transmittance spectra of EC devices using a-NiO_x_ (orange trace) and c-NiO_x_ (black trace) as counter electrodes at colored and bleached states. The spectra were recorded after coloring at −2.1 V or bleaching at +2.1 V for 60 s. The images are the solid-state device using a-NiO_x_ as counter electrode in bleached and colored states. (B) Transmittance change at wavelength of 633 nm for the EC devices with a-NiO_x_ (orange trace) and c-NiO_x_ (black trace) on FTO as counter electrode as a function of time. The devices were bleached at +2.1 V for 30 s, then colored at −2.1 V for another 30 s. Switching times t_color_ and t_bleach_ are indicated. (C) Changes in optical density of the devices using a-NiO_x_ and c-NiO_x_ (inset) on FTO as counter electrode at 633 nm as a function of charge density. Coloration efficiency values were determined by fitting the linear region of the plot (Equation 1 and 2). See also Figures S5, S8, and S9.

The EC performance of the full devices containing either a- or c-NiO_x_ counter electrode was tested by using UV-Vis spectroscopy to measure the optical properties under applied potentials (Figure 3). The optical modulation (ΔT_633 nm_) was determined by recording the transmittance of the devices in either a fully colored or a fully bleached state at potentials of −2.1 or +2.1 V, respectively. EC devices with the a-NiO_x_ counter electrode showed a ΔT_633 nm_–60%, compared with ΔT_633 nm_–26% for the device with c-NiO_x_ counter electrode (Figure 3A) (*c.f*. 19% for devices in which the counter electrode was bare FTO [Figure S5]). Switching times from the fully colored to the fully bleached state (t_bleach_), and the reverse (t_color_), were measured by tracking the transmittance at λ = 633 nm in response to consecutive applied potentials of +2.1 and −2.1 V for 30 s each (Figure 3B). The device with the a-NiO_x_ counter electrode showed rapid switching times of t_bleach_ ∼ 6 s and t_color_ ∼4 s, whereas the device with a c-NiO_x_ counter electrode showed much slower t_color_ and t_bleach_ of 78 and 17 s, respectively.

CE, which measures the change in optical density acquired by injection of charge per unit area, was determined in accordance with Equations 1 and 2:

(Equation 1) $$\mathrm{CE}=\frac{\Delta \left(\text{OD}\right)}{\Delta \text{Q}}$$

(Equation 2) $$\Delta \left(\text{OD}\right)=\log\left(\frac{T_{b}}{T_{c}}\right)$$

where Δ(OD) is the change in optical density and ΔQ is the charge density (C/cm^2^) obtained from electrochemical measurements; T_b_ is the maximum transmittance in the bleached state at a fixed wavelength (obtained from Figure 3B); and T_c_ is the varying transmittance obtained during the coloration process at this same wavelength. The CE values derived from the slope of the linear region of Δ(OD) versus ΔQ (Figure 3C) were determined to be 141 cm^2^/C for a device containing an a-NiO_x_ counter electrode. This value is approximately twice that of the device with c-NiO_x_ counter electrode (72 cm^2^/C).

The differences in performance metrics of the EC devices containing either an a-NiO_x_ or c-NiO_x_ counter electrode demonstrate the superior EC performance of a-NiO_x_ systems. It appears that the superior performance of a-NiO_x_ is manifest in the superior ion-storage properties of the films given that a-NiO_x_ is characterized by a charge capacity of 4.1 mC/cm^2^ during the pre-lithiation process that is 2-fold higher than the value measured for c-NiO_x_ (1.6 mC/cm^2^). These findings are consistent with the higher ion storage capacity generally observed for amorphous materials (Ku et al., 2012, Lee et al., 2014). The improved ion storage performance that arises in amorphous materials is believed to be due to the larger interstitial spaces between more disordered atoms (Lee et al., 2014, Legrain et al., 2015, Llordés et al., 2016). These larger interatomic spaces can accommodate intercalated ions and facilitate the mobility of ions in materials. In the case of NiO_x_ counter electrode materials in EC devices, the amorphous film is capable of balancing more charges during each EC cycle, thereby accommodating greater lithium insertion into the WO_3_ during each coloration cycle and resulting in higher ΔT. Furthermore, the enhanced ion mobility in a-NiO_x_ yields faster switching times between bleach and colored states.

Table 1 lists the performance of our solid-state EC devices containing photodeposited NiO_x_ counter electrodes along with other reported devices of similar sizes and configurations. The ΔT_633 nm_ of 60% reported here for the a-NiO_x_ is in keeping with systems synthesized by other methods. The switching times (t_bleach_ and t_color_) of our device is among the fastest reported to date, exceeded only by t_bleach_ for films made using high-vacuum sputtering methods. The CE of 141 cm^2^/C exceeds all other known examples of devices based on WO_3_ and NiO_x_ materials, highlighting the excellent EC performance of our solution-processed devices containing amorphous films. It is worth noting that the performance is also comparable with commercially available EC windows, which use sputtered WO_3_ and NiO_x_ films and polymer-based electrolytes. These full-scale windows typically show an optical modulation of merely ∼45% over the visible light region and a switching time of 15–20 min (with the important caveat that the device sizes are different) (Range of products, 2018).

### Performance Dependence on a-NiO_x_ Thickness

**Figure 4:**
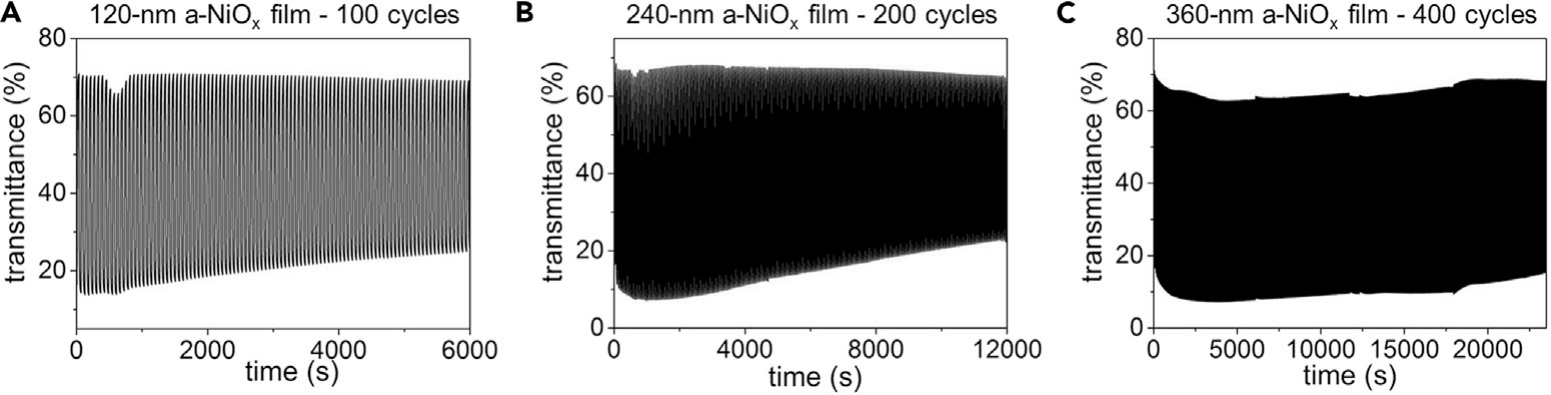
Thickness-Dependent Electrochromic Cycling Stability (A–C) Transmittance changes at wavelength of 633 nm as a function of time during the electrochromic switching between colored and bleached states for EC devices using a-NiO_x_ films with thicknesses of (A) 120 nm, (B) 240 nm, and (C) 360 nm as counter electrodes. In each electrochromic cycle, the devices were held at potential −2.1 V for 30 s then switched to +2.1 V for another 30 s. See also Figure S8.

The thicknesses of the a-NiO_x_ films are controlled simply by spin casting and UV treating a variable number of NiCl_2_ precursor layers on the FTO substrate before annealing. NiO_x_ films with thicknesses of 70, 120, 240, and 360 nm were prepared by 3, 5, 10, or 15 layers of deposition, respectively (Figures 2, S6, and S7). Amorphous NiO_x_ films of increasing thicknesses (120, 240, and 360 nm) were assembled into EC devices as the counter electrode material (with all other parameters held constant) and similarly assessed for their optical properties in response to an applied voltage. Increasing thickness of a-NiO_x_ from 120 nm to 360 nm caused a slight decrease in ΔT_633 nm_ from 60% to 56% (Figure S8). Switching times t_bleach_ and t_color_ also increased slightly reaching 11 and 13 s, respectively, for the thickest of the three a-NiO_x_ films (Figure S8). The stability of the EC devices was also tested by tracking the transmittance change over time while continuously switching between the colored and bleached states. The thicker NiO_x_ films exhibit enhanced cycling stability despite the loss in optical modulation and increase in switching times. SEM characterization showed that the 120-nm a-NiO_x_ film was compromised during cycling, whereas the morphologies of the thicker films (240 and 360 nm) were mostly maintained (Figure S9). For the device with a 120-nm thickness of a-NiO_x_, 81% of the initial optical modulation value remained after 100 cycles (Figure 4A). The EC device with a 240-nm thickness of a-NiO_x_ retained 90% of the initial ΔT after 100 cycles and 75% after 200 cycles (Figure 4B). Further increasing the thickness of the a-NiO_x_ film to 360 nm enabled the device to retain nearly 100% of the initial ΔT after 400 cycles (Figure 4C). By contrast, solid-state devices using inkjet-printed WO_3_ and NiO nanoparticle electrodes retain only 80% ΔT_633 nm_ after 100 cycles, with significant further degradation thereafter (Cai et al., 2016a).

### Conclusion

Solution-based photodeposition of readily available metal precursors (WCl_6_ and NiCl_2_) can be used to synthesize both the working and counter electrode materials of EC windows. The exceptional performance of EC devices containing these materials is demonstrated here with amorphous WO_3_ and NiO_x_ thin films assembled together with a PMMA-based gel electrolyte. The resulting solid-state systems exhibit optical modulations (ΔT_633 nm_), switching times (t_color_ and t_bleach_), coloration efficiencies (CE), and cycling stabilities commensurate with the best reported devices to date, including those produced by expensive or specialized methods. We also show that this low-temperature process yields amorphous layers of NiO_x_ that act as superior counter electrodes relative to crystalline NiO_x_, which we anticipate will trigger greater research efforts on the use of amorphous materials for EC devices. These collective results represent an opportunity to drive down the costs of energy-saving EC windows that have already attracted substantial commercial investment.

### Limitations of the Study

This study demonstrates the ability to use photodeposition to make uniform EC nickel and tungsten oxide layers. The formation of the photodeposited a-NiO_x_ films must be optimized to compete with sputtering at an industrial scale. Future investigations will also seek to resolve the factors that lead to the increases in transmittance of the bleached state during cycling (Figure 4C).

## Methods

All methods can be found in the accompanying Transparent Methods supplemental file.

## Supplemental Information

Document S1. Transparent Methods and Figures S1–S9
