# Supplementary material for: Solution-Deposited Solid-State Electrochromic Windows
Source: iScience. 2018 Nov 10;10:80–6. doi: 10.1016/j.isci.2018.11.014 (PMC6277218; doi:10.1016/j.isci.2018.11.014)
Supplement: Document S1. Transparent Methods and Figures S1–S9 [file mmc1.pdf]

ISCI, Volume *10*

Supplemental Information

Solution-Deposited Solid-State

Electrochromic Windows

Wei Cheng, Marta Moreno-Gonzalez, Ke Hu, Caroline Krzyszkowski, David J. Dvorak, David M. Weekes, Brian Tam, and Curtis P. Berlinguette

Supplemental Figures

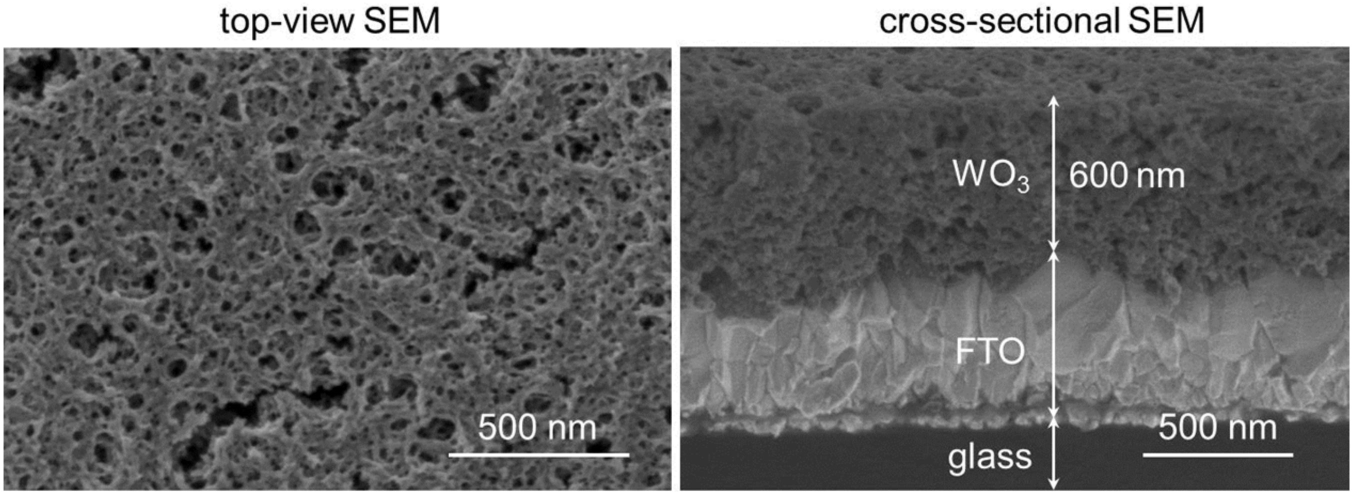

Figure S1. SEM characterization of a-WO_3 films, Related to Figure 2. Top-view (left) and cross-sectional (right) SEM images of 5-layer WO_3 films produced by photodeposition followed by annealing at 100°C for 1 hr. Cross-sectional SEM images were acquired on fresh edges of cleaved samples at a tilt angle of 52 degrees.

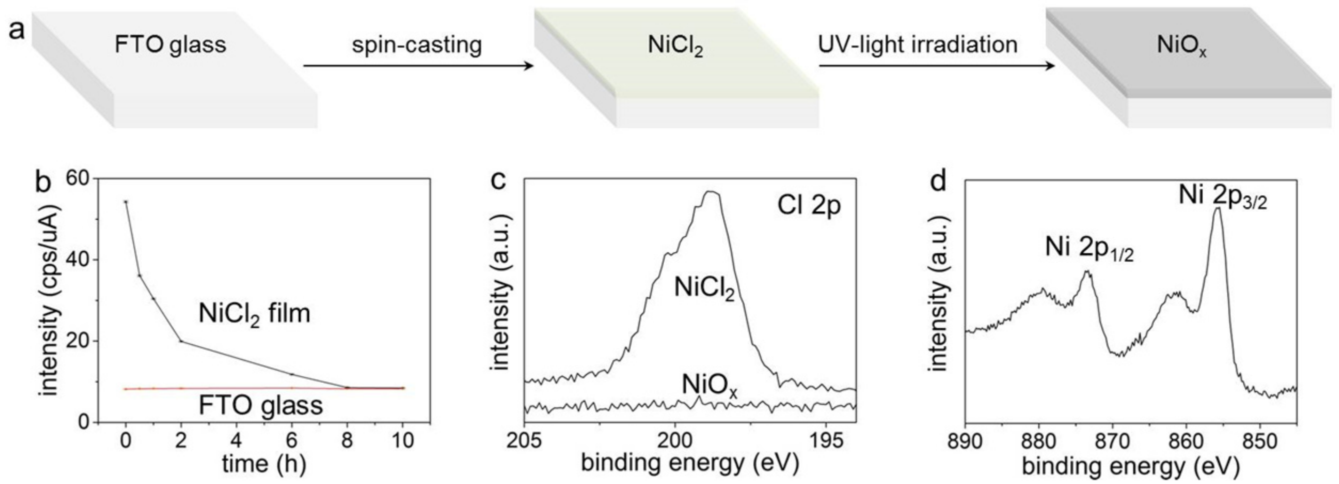

Figure S2. Photodeposition of NiO_x films, Related to Figure 2. (a) Schematic illustration showing UV light irradiation of spin-cast $NiCl_2$ precursor films on FTO glass leads to formation of NiO_x. (b) Content of Cl in precursor film determined by X-ray fluorescence (XRF) analyzer as a function of UV irradiation time. The chloride ions are completely removed by UV light illumination in 8 hrs. (c) XPS spectra of $NiCl_2$ precursor and as-formed NiO_x in binding energy range corresponding to Cl 2p. No Cl 2p signal exists in as-formed NiO_x, confirming complete decomposition of chloride ions by UV irradiation. (d) XPS spectrum of NiO_x in the binding energy range corresponding to Ni 2p, matching well with that of Ni^{2+}.

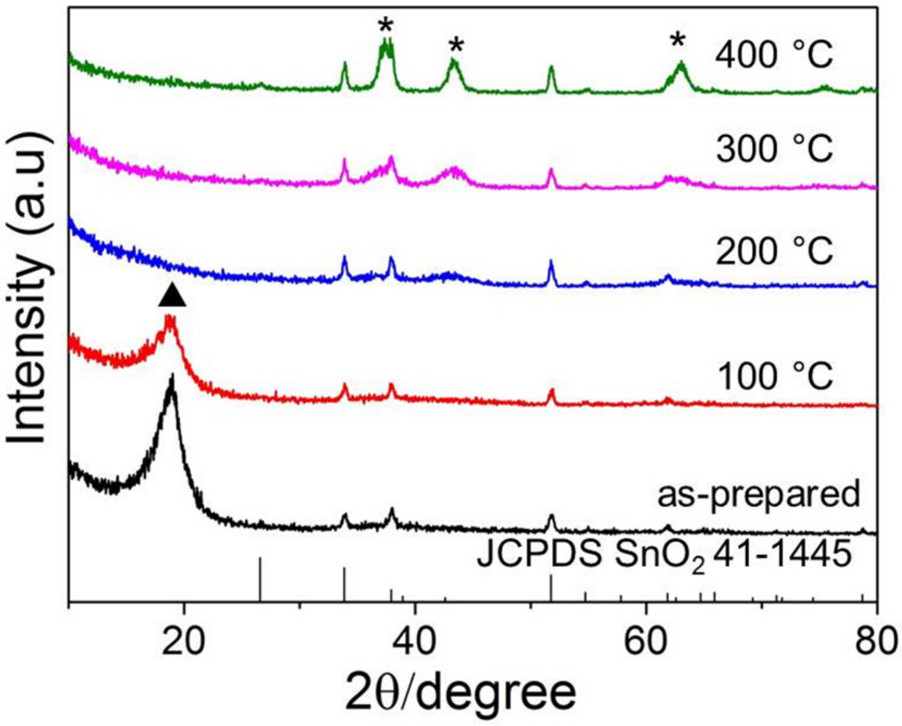

Figure S3. XRD characterization, Related to Figure 2. XRD patterns of photodeposited NiO_x films annealed at 100, 200, 300, and 400°C for 1 hr. Broad diffraction peak at labeled with ▲ symbol corresponds to (100) planes of α-$Ni(OH)_2$. The reflections denoted with * symbol correspond to cubic phase NiO (JCPDS 47-1049). All other reflections arising from the FTO substrate can be indexed to SnO_2 (JCPDS 41-1445).

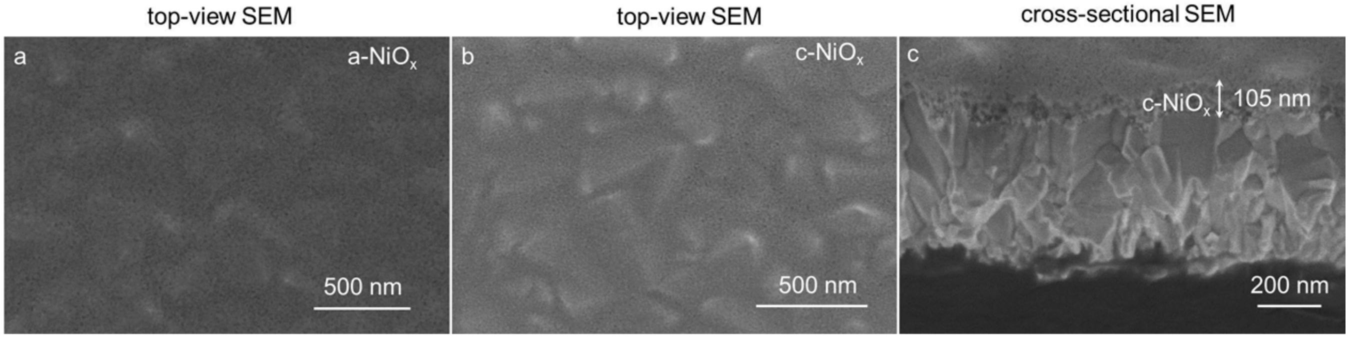

Figure S4. SEM characterization of amorphous and crystalline NiO_x films, Related to Figure 2. (a, b) Top-view and (c) cross-sectional SEM images of a- and c-NiO_x films produced by photodeposition followed by annealing at 200 and 400°C for 1 hr, respectively. Cross-sectional SEM images were acquired on fresh edges of cleaved samples at a tilt angle of 52 degrees.

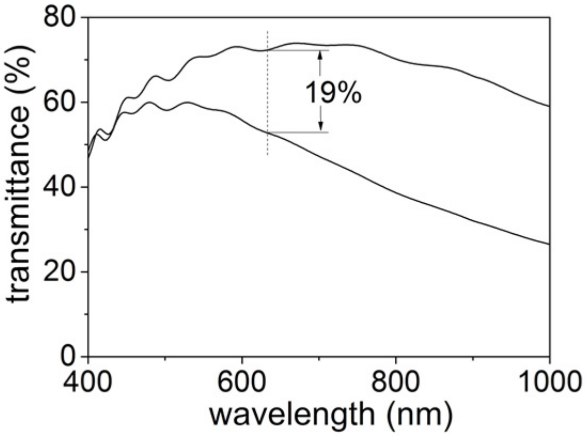

Figure S5. Optical modulation of an EC device with bare FTO counter electrode, Related to Figure 3. Transmittance spectra of EC device using bare FTO as the counter electrode in the colored and bleached states. The spectra were recorded after coloring at -2.1 V or bleaching at +2.1 V for 60 s. $\Delta T_{633\ nm}$ was determined to be 19%.

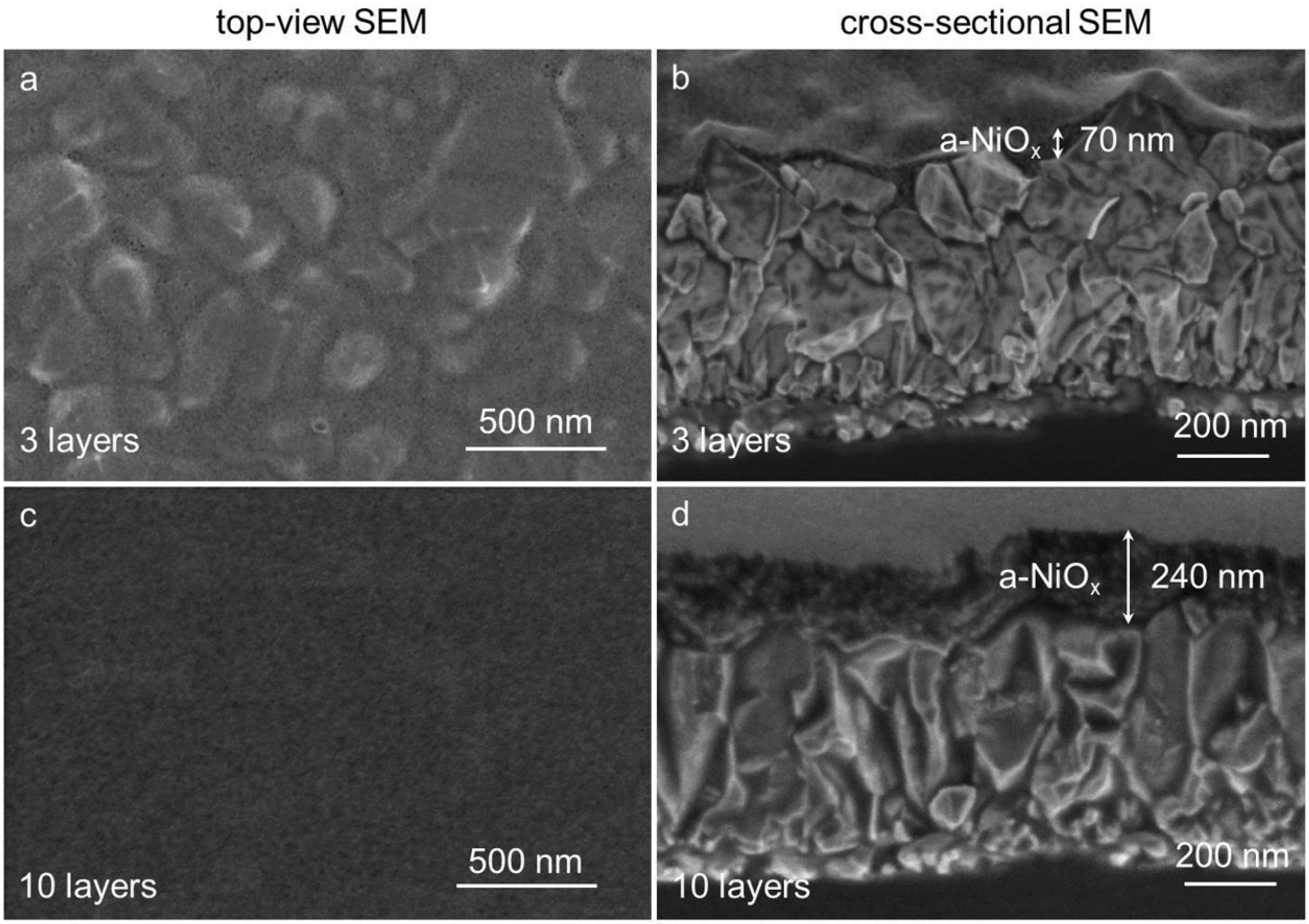

Figure S6. SEM characterization of a-NiO_x film, Related to Figure 2. (a, c) Top-view and (b, d) cross-sectional SEM images of 3-layer and 10-layer a-NiO_x films produced by photodeposition followed by annealing at 200°C for 1 hr. Cross-sectional SEM images were acquired on fresh edges of cleaved samples at a tilt angle of 52 degrees.

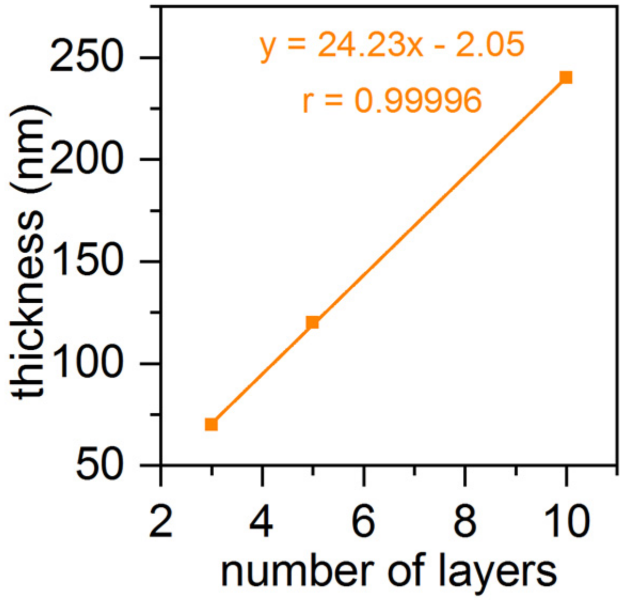

Figure S7. Relation between film thickness and number of deposition layers, Related to Figure 2.
A linear relation was found between the thickness of a-NiO_x films determined from cross-sectional SEM images (see Figures 2 and S6) and the number of layers of a-NiO_x coated on FTO substrates.

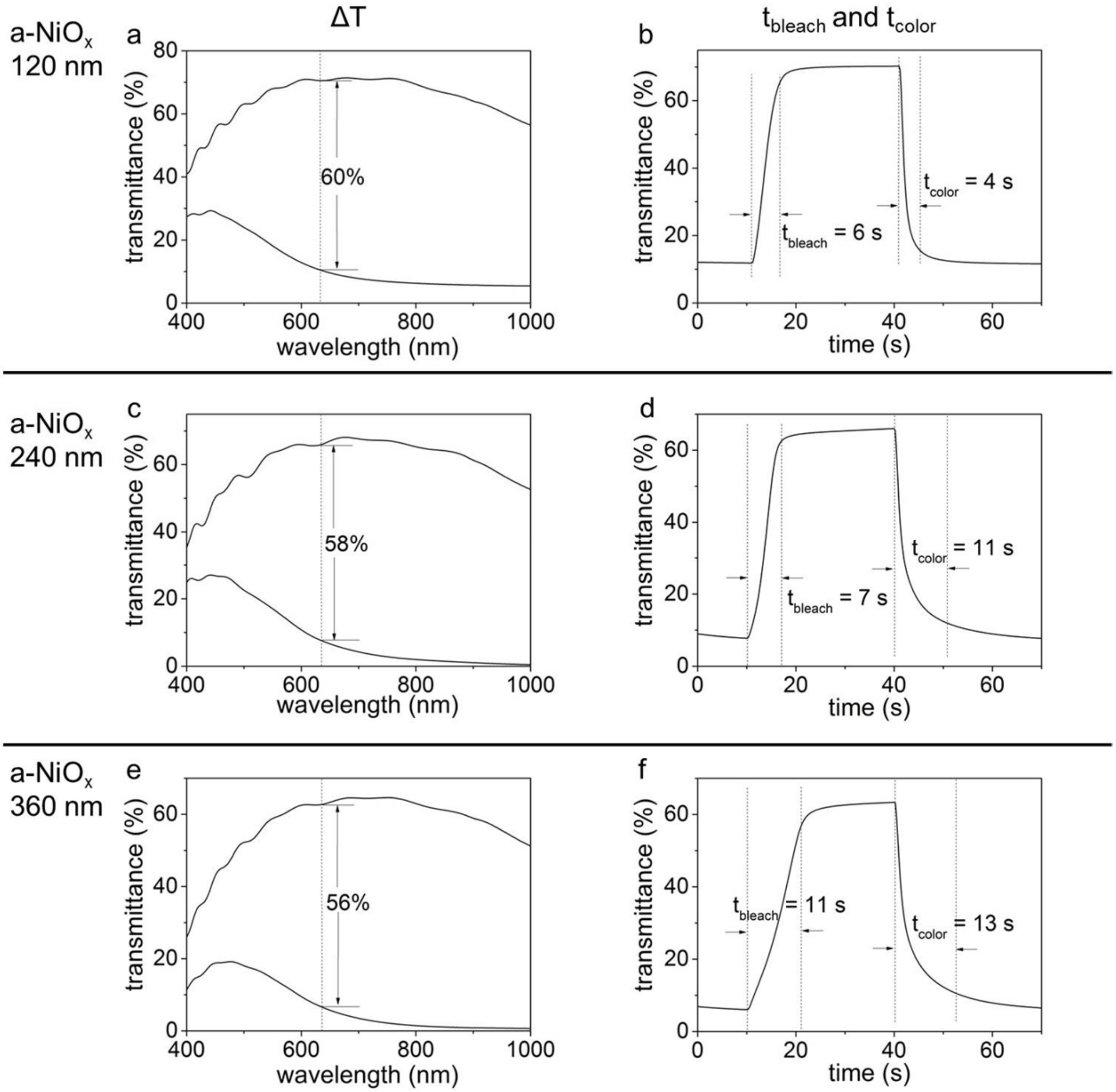

Figure S8. Thickness-dependent electrochromic performance, Related to Figures 3 and 4. Optical modulation and switching times of devices using a-NiO_x films with thicknesses of (a, b) 120 nm, (c, d) 240 nm, and (e, f) 360 nm as counter electrode materials. The 360-nm a-NiO_x film was made by 15 layers of coating and the thickness was calculated from the equation shown in Figure S7.

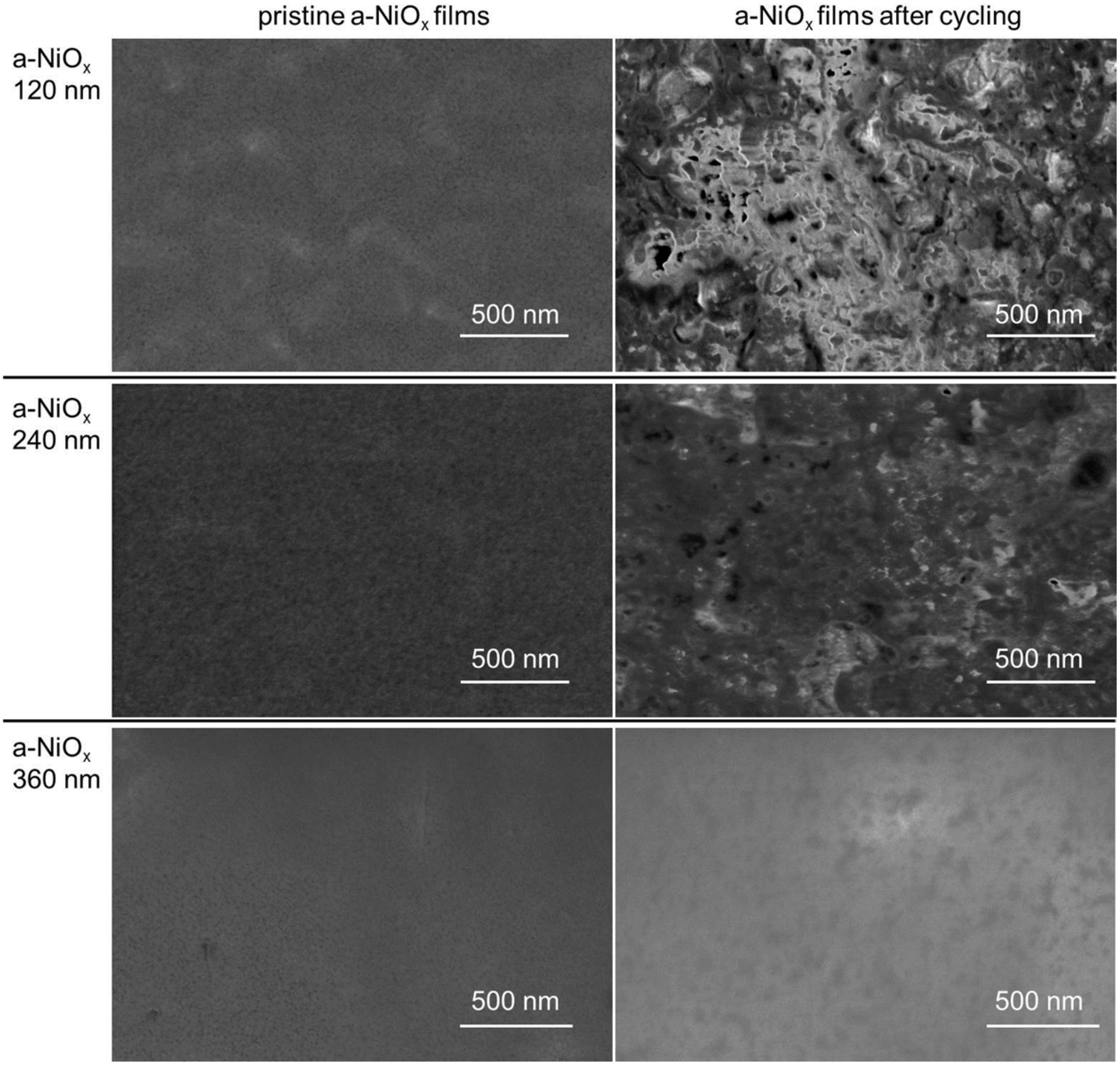

Figure S9. Influence of electrochromic cycling on the morphologies of a-NiO$_x$ films, Related to Figure 4. SEM images of a-NiO$_x$ films with different thicknesses before and after electrochromic cycling.

Transparent Methods

Materials

WCl_6 (99.99%), $NiCl_2 \cdot 6H_2O$ (99.9%), $LiClO_4$ (99.99%), PC (99.7%, anhydrous), 2-propanol (99.9%), and PMMA (M_w ~ 350000) purchased from Sigma-Aldrich were used directly without further purification. FTO-coated glass purchased from Hartford Glass (TEC7) was first cleaned by sequential ultrasonication in detergent solution (Extran 300, EMD), distilled water, acetone, and isopropanol, and then it was dried in N_2 flow and further subjected to UV-Ozone treatment for 30 min prior to use.

Film syntheses

In a typical synthesis of NiO_x film, a 0.25 M $NiCl_2$ aqueous solution prepared by dissolving 0.24 g (1.0 mmol) $NiCl_2 \cdot 6H_2O$ in 4 ml distilled water was spin-coated on FTO glass at 3000 rpm for 60 s (Laurell model WS-650MZ-23NPP-Lite). The resultant precursor thin films were subjected to UV (Atlantic Ultraviolet G18T5VH/U; λ_{max} = 185 nm) irradiation until complete decomposition was confirmed by tracking the chlorine content by XRF analysis. To produce multi-layer thin films, the spin-coating and UV light irradiation steps were repeated multiple times. The as-deposited films were annealed in an oven (Ney Vulcan 3-550) in air at different temperatures for 1 hr using a ramping rate of 10 °C/min.

The same procedures were used for synthesis of a-WO_3 films, except that a 0.25 M WCl_6 solution in isopropanol was used as precursor solution and UV irradiation time was 5 min. The as-deposited films were annealed in air at 100°C for 1 hr. WO_3 films with 5 layers of deposition were prepared and used in all devices throughout this work.

Electrolyte preparation

$LiClO_4$ and PMMA were dried at 100°C overnight, while PC was dried using a molecular sieve type-3A with a w/v of 20% overnight. The molecular sieves were activated by annealing at 300°C overnight before use. 0.53 g $LiClO_4$ was dissolved in 10 ml dry PC to form a 0.5 M solution. 1.34 g PMMA was then added to the $LiClO_4$-PC solution under magnetic stirring. The mixture was stirred and heated at 60°C on a hot plate for 16 hrs to form a colorless transparent gel.

Device assembly

Prior to device assembly, NiO_x films were prelithiated by electrochemical means using a conventional three electrode system with NiO_x films on FTO glass as the working electrode, Ag/AgCl as the reference electrode, Pt wire as the counter electrode and 1 M $LiClO_4$-PC as electrolyte. The lithium ions were intercalated into the NiO_x films by applying a potential of -1.5 V for 5 min.

Electrochromic devices were fabricated by placing a 2 × 2 cm square silicone rubber sheet (50 A, thickness = 1 mm; McMaster-CARR) with a centered hollow circle (diameter = 1.6 cm) on top of the prelithiated NiO_x film on FTO glass that serves as the counter electrode. The $LiClO_4$-PC-PMMA gel electrolyte was then drop-cast into the hollow circle. The FTO glass coated with a-WO_3 films (working electrode) was then laid on top of the silicon spacer to form a closed cell. Epoxy glue was used to seal the cell. The assembled devices were then heated at 60°C overnight before measuring the electrochromic performance. The assembled device had an active area of 2.0 cm^2.

Physical methods

An XRF analyzer (Thermo Fisher Scientific) was used to track chlorine in thin films. XPS analyses were carried out on a Leybold MAX200 spectrometer using Al Kα radiation. The pass energy was 192 eV for the survey scan and 48 eV for the narrow scan. Grazing incidence X-ray diffraction (GIXRD) experiments were performed with a Rigaku Smartlab diffractometer in parallel beam mode using Cu Kα radiation. Data were collected with a scan step of 0.04°, an incidence angle of 0.3°, and a scan rate of 3° min^{-1}. SEM images were acquired in secondary electron mode at 1 kV accelerating voltage on a Helios NanoLab 650 Focused Ion Beam SEM. To obtain cross-sectional images, FTO substrates coated with a-NiO_x films were cleaved in-house and the freshly broken edges were imaged at a tilt angle of 52°. The electrochromic properties of EC devices were measured with a PerkinElmer Lambda 35 UV-Vis spectrophotometer and a CHI660D potentiostat.
